# Five days of bed rest in young and old adults: Retainment of skeletal muscle mass with neuromuscular electrical stimulation

**DOI:** 10.14814/phy2.16166

**Published:** 2024-08-18

**Authors:** Sofie K. Hansen, Pernille Hansen, Hanne Nygaard, Hans D. Grønbæk, Tania W. Berry, Camilla M. Olsen, Per Aagaard, Lars G. Hvid, Jakob Agergaard, Flemming Dela, Charlotte Suetta

**Affiliations:** ^1^ Geriatric Research Unit Copenhagen University Hospital ‐ Bispebjerg and Frederiksberg Copenhagen Denmark; ^2^ CopenAge, Copenhagen Center for Clinical age Research University of Copenhagen Copenhagen Denmark; ^3^ Department of Emergency Medicine Copenhagen University Hospital ‐ Bispebjerg and Frederiksberg Copenhagen Denmark; ^4^ Department of Sport and Clinical Biomechanics University of Southern Denmark Odense Denmark; ^5^ Exercise Biology, Department of Public Health Aarhus University Aarhus Denmark; ^6^ The Danish MS Hospitals, Ry and Haslev Haslev Denmark; ^7^ Department of Orthopedic Surgery, Institute of Sports Medicine Copenhagen Copenhagen University Hospital—Bispebjerg and Frederiksberg Copenhagen Denmark; ^8^ Department of Clinical Medicine, Center for Healthy Aging University of Copenhagen Copenhagen Denmark; ^9^ Xlab, Department of Biomedical Sciences University of Copenhagen Copenhagen Denmark; ^10^ Department of Physiology and Biochemistry Riga Stradins University Riga Latvia

**Keywords:** aging, atrophy, disuse, muscle strength

## Abstract

The consequences of short‐term disuse are well known, but effective countermeasures remain elusive. This study investigated the effects of neuromuscular electrical stimulation (NMES) during 5 days of bed rest on retaining lower limb muscle mass and muscle function in healthy young and old participants. One leg received NMES of the quadriceps muscle (3 × 30min/day) (NMES), and the other served as a control (CON). Isometric quadriceps strength (MVC), rate of force development (RFD), lower limb lean mass, and muscle thickness were assessed pre‐and post‐intervention. Muscle thickness remained unaltered with NMES in young and increased in old following bed rest, while it decreased in CON legs. In old participants, mid‐thigh lean mass (MTLM) was preserved with NMES while decreased in CON legs. In the young, only a tendency to change with bed rest was detected for MTLM. MVC and early‐phase RFD decreased in young and old, irrespective of NMES. In contrast, late‐phase RFD was retained in young participants with NMES, while it decreased in young CON legs, and in the old, irrespective of NMES. NMES during short‐term bed rest preserved muscle thickness but not maximal muscle strength. While young and old adults demonstrated similar adaptive responses in preventing the loss of skeletal muscle thickness, RFD was retained in the young only.

## 1 INTRODUCTION

Skeletal muscles not only impact locomotive abilities but also affect the regulation of body metabolism (Stump et al., 2006). Consequently, deterioration or loss of muscle mass and function can negatively affect health (García‐Hermoso et al., 2018; Mayer et al., 2020; Wang et al., 2020). Short‐term disuse, defined as sustained physical inactivity for less than 7 days, can alter the contractile and metabolic characteristics of skeletal muscle, leading to muscular atrophy and impairments in mechanical muscle function (Mulder et al., 2015; Reidy et al., 2017; Suetta et al., 2012; Tanner et al., 2015; Wall et al., 2014). The latter involves reductions in maximal isometric and dynamic muscle strength, accompanied by attenuated levels of rate of force development (RFD) in the initial contraction phase of rising muscle force (Deschenes et al., 2008; Hvid et al., 2013, 2014; Suetta et al., 2012).

Furthermore, the consequences of skeletal muscle atrophy as a result of short‐term disuse go beyond physical weakness as such; evidence suggests that the absence of contractile activity during periods of disuse contributes to impairments in postprandial glucose disposal, thereby reducing insulin sensitivity and affecting overall metabolic homeostasis (Crossland et al., 2019; Mikines et al., 1991; Reidy et al., 2018).

The effect of short‐term disuse on muscle function and morphology has previously been investigated in both young and old adults, yielding divergent results. Studies have suggested that older adults lose more muscle mass (Reidy et al., 2018; Tanner et al., 2015) and demonstrate larger decrements in muscle strength and RFD than younger adults, (Hvid et al., 2014; Tanner et al., 2015). In contrast, others have reported similar magnitude of disuse‐induced declines in muscle strength and muscle mass between young and old adults (Deschenes et al., 2008; Hvid et al., 2014; Suetta et al., 2012). These results suggest an incomplete insight into the physiological and functional impacts of short‐term disuse between young and old adults, emphasizing the need for experimental setups allowing direct comparisons of individuals of different ages.

To mitigate the adverse effects of short‐term disuse on muscle mass and contractile function, it appears essential to explore strategies to engage muscle activity during periods of sustained immobilization. While exercise training can offset losses in muscle mass and strength during periods of muscle disuse (Bamman et al., 1998; Oates et al., 2010), not all patients can exercise during acute illness or recovering from bone fractures or ligament injury. Therefore, Neuromuscular Electrical Stimulation (NMES) represents a promising counteractive strategy, by inducing contractile muscle activity, thereby potentially counterbalancing the disuse‐induced loss in muscle function and mass, respectively (Blazevich et al., 2021; Maffiuletti et al., 2023). In support of this notion, NMES has shown efficacy in preserving muscle mass among various populations when exposed to short‐term immobilization, involving both healthy individuals (Dirks et al., 2014; Reidy et al., 2017) and various patient groups (Alqurashi et al., 2023; Dirks et al., 2015; Karlsen et al., 2020). Yet, a gap exists in the knowledge about the loss of muscle mass and the impairment in mechanical muscle function evoked by short‐term disuse and compared across young and older adults.

The purpose of the present study, therefore, was to investigate the effect of daily sessions of electrically evoked muscle contractions (NMES) during 5 days of sustained bed rest on the preservation of lower limb skeletal muscle mass and mechanical muscle function, respectively, in young versus old adults. It was hypothesized that lower‐limb NMES would counteract the detrimental effects of bed rest, leading to attenuated losses in muscle mass and mechanical muscle function (strength, RFD) compared to CON legs after 5 days of bed rest. Moreover, we anticipated that young and old adults would respond similarly to both bed rest alone and bed rest with concurrent NMES, respectively.

## 2 MATERIALS AND METHODS

### 2.1 Ethical approval

The study was approved by the Ethical Committee of the Capital Region Copenhagen (H‐20038614) and registered on clinicaltrials.gov (NCT05617222). All procedures of the study conformed to the Declaration of Helsinki and written informed consent was obtained from all participants.

### 2.2 Participants

Thirty‐two healthy young (18–30 years of age) and old (65–80 years of age) male and female adults (50/50%) were included. Before inclusion, potential participants were screened for eligibility to exclude individuals with diseases affecting the musculoskeletal system or contraindicated participation in strict bed rest (e.g., diabetes, cardiovascular or neural diseases, etc.), use of anabolic steroids or medications affecting skeletal muscle tissue or myofibrillar protein synthesis.

### 2.3 Protocol overview

**FIGURE 1:**
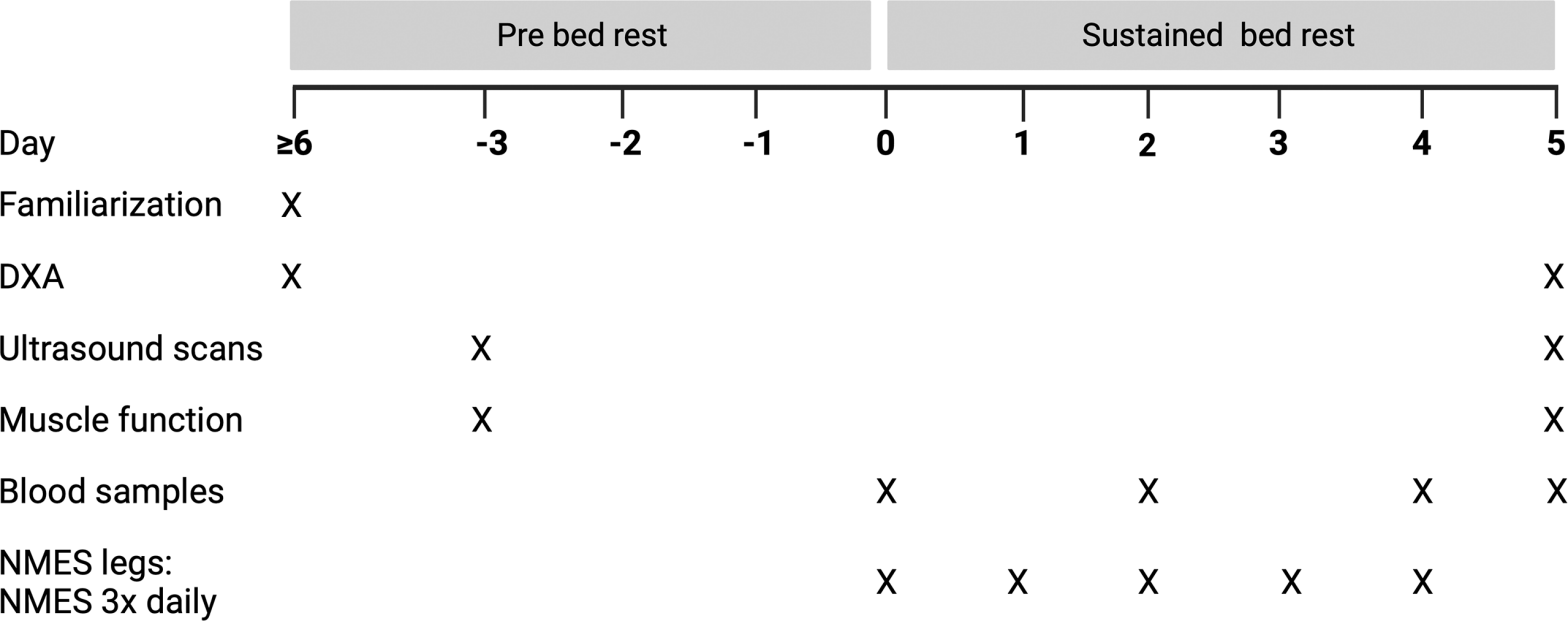
Schematic illustration of the study timeline. Prior to the bed rest period, young (*n* = 16) and old (*n* = 16) healthy participants were included and familiarized to testing procedures. Following this, on Day −3, participants were pre‐intervention tested. On Day 0, participants arrived in the fasting state in the morning where blood samples were collected, and hereafter the bed rest was initiated. Throughout the 5 days of bed rest, NMES leg received 3 × 30 min NMES. The last NMES session was in the evening on Day 4. On Day 5, blood samples were drawn in the fasted state in the morning, following which participants were transported by wheelchair to the Lab for post‐intervention testing.

Following inclusion, participants were familiarized with all testing procedures (Figure 1). Three days before the bed rest period, participants re‐visited the Lab for pre‐intervention testing. After pre‐testing, an accelerometer (SENS motion, SENS Aps, Denmark) was placed on the participant's leg (laterally on the thigh, 10 cm above the femur condyle) to monitor habitual activity levels.

On Day 0, participants arrived at the Lab after an overnight fast, where blood samples were collected from an antecubital vein and were subsequently directed to the hospital ward, where the bed rest was to take place. Stratified by gender and age, the participants' dominant and non‐dominant legs were randomly allocated to NMES (bed rest + NMES) or CON (control, bed rest only). Post‐testing was performed on Day 5 (Figure 1), approximately at the same time of day and by the same investigator as at pre‐intervention testing.

All test procedures were performed unilaterally in the order of the dominant leg followed by the non‐dominant leg. Participants were asked to avoid any fatiguing physical activities 48 h before pre‐intervention testing and to refrain from any vigorous activity 24 h before the test session.

### 2.4 Bed rest procedures

Participants were instructed to spend all their time in bed throughout the 5‐day bed rest period but were allowed to be in a seated position while eating, reading, using a laptop/smartphone, knitting, and performing similar activities. Hygiene and sanitary activities were performed in bed or by use of a wheelchair. Before commencing the bed rest, participants received instructions on how to transfer from the bed to the wheelchair without standing up or in other ways using the legs.

The adherence to sustained bed rest during the 5‐day unloading protocol was monitored by research staff and by accelerometer recording. From the accelerometers, time spent lying down and intensity of movements were registered and controlled throughout the bed rest period.

Participants consumed a standardized isocaloric diet, delivered by the hospital kitchen. The diet was matched to fit participants' reduced physical activity levels during bed rest, equaling resting energy expenditure and ensuring an adequate intake of protein of approximately 1 g/protein/kg body weight (BW) was administered daily over three main meals. Total caloric intake was calculated by use of the Harris–Benedict equation (modified by Wang et al., 2000, their equation 6):

REE=21.7×FFM+374

REE is the resting energy expenditure and FFM is the fat‐free mass.

Following the 5 days of bed rest (Figure 1), participants were passively transported to the test Lab using a wheelchair, where all tests and examinations were repeated, starting with drawing of blood samples and ultrasound imaging before being allowed to stand again and perform the remaining tests (more details provided below).

### 2.5 NMES protocol

During the bed rest period, one leg received neuromuscular electric stimulation (NMES) of the knee extensors three times daily, just before or after a meal (breakfast, lunch, dinner) while administered and supervised by on‐site research personnel.

In brief, the knee extensor muscles were stimulated using an electrical muscle stimulator (EMP 4 ECO+, 101,060, Schwa‐medico, Germany) equipped with biocompatible stimulation electrodes (Merhi et al., 2023). The electrodes were placed over the proximal and distal part of the thigh, allowing a long inter‐electrode distance.

The neuromuscular electrical stimulation (NMES) protocol comprised a 2‐min *warm‐up phase* with a frequency of 6 Hz and pulse width of 300 μs. This was followed by a 30‐min *work phase* where the electrical stimulation was delivered using biphasic current stimulation to the muscle belly, with a frequency of 60 Hz and pulse width of 400 μs. Each stimulus train lasted 7 s with a gradual rise and fall in intensity (ramp up/ramp down: 1‐s rise, 5‐s contraction, 1‐s relaxation) followed by 10 s of passive rest. Stimulus intensity (amplitude, mA) was increased until muscular contractions were palpable and visible, and the participant would identify the intensity as perceptible yet tolerable. An increase in NMES intensity throughout stimulation sessions was allowed, as the tolerance to stimulation increased both intra‐session and inter‐session. The intensity was recorded in each session.

## 3 MEASUREMENTS

### 3.1 Blood samples

On Days 0 and 5, blood samples were collected in a fasted state for subsequent analysis at the Clinical Biochemical Department at the hospital, mirroring the comprehensive blood test panel routinely conducted on hospitalized patients to obtain an overview of various health‐related variables. Moreover, blood samples were collected in a fasted state in the morning on Days 0, 2, 4, and 5. Blood was collected in 9 mL tubes with EDTA, kept on ice for min. 10 to max. 30 min, and subsequently centrifuged at 4000 RPM (3172×*g*) for 10 min, before plasma was aliquoted to Eppendorf tubes and stored at −80°C until further analysis.

Plasma glucose, insulin, C‐peptide and cortisol concentrations were analyzed using commercially available kits (glucose [GLUC2], reference 04657527190, cobas; Elecys Insulin, reference 12,027,547, cobas; C‐peptide, reference 03184897 190, cobas; cortisol [Elecsys Cortisol II], reference 06687733190, cobas).

Homeostasis model assessment of insulin resistance (HOMA IR‐index) was calculated from fasting plasma glucose and insulin concentrations using HOMA calculator software (HOMA2 Calculator v2.2.4).

### 3.2 Lean mass (dual‐energy x‐ray absorptiometry)

Whole body dual‐energy x‐ray absorptiometry (DXA) scans (Lunar DPX‐IQ DEXA scanner, GE Healthcare, Chalfont St. Giles, UK) were performed before and following bed rest.

Participants were asked to visit the toilet before being placed in a supine position in the scanner according to a standard protocol: Heels were separated by a 22 cm block and legs were kept in place by using a strap. Arms were placed with hands aligned with the hips and thumbs pointing upwards. Leg muscle mass was segmented using the standard software package (Lunar iDXA Forma enCORE vs.15) by a blinded investigator.

Mid‐thigh lean mass (MTLM) was computed with reference to a 4 cm calibration box at 50% femur length (i.e., 2 cm above and 2 cm below 50% femur length) placed between the lateral femur condyle and superior part of the trochanter major (Norheim et al., 2017). Identical ROIs (region of interest) were assigned at pre‐ and post‐scans in the same participant.

### 3.3 Muscle thickness (ultrasound scans)

Sagittal ultrasound images of the vastus lateralis muscle were recorded with a conventional real‐time US scanner (Philips iU22, Bothell, WA, USA) with a 7.5‐MHz linear array transducer. Images were obtained in the seated position (90° flexion in the hip and knee joint) at 50% of femur length over the mid‐belly of the vastus lateralis muscle (Suetta et al., 2008).

The specific scan position was marked on the frontal thigh with a surgical permanent marker to ensure identical scan positions at pre‐and post‐intervention. Two images were obtained from each participant. The best (most clear) image was chosen for further analysis and hereafter analyzed for VL muscle thickness three times (ImageJ2, version 2.14.0/1.54f; National Institute of Health, Bethesda, MD) (Suetta et al., 2009) by a blinded investigator. Mean values were recorded.

### 3.4 Muscle strength assessment

Maximal knee extensor muscle strength and RFD were assessed as maximal voluntary isometric contractions (MVC) in a custom‐built dynamometer (Suetta et al., 2009), constructed in the form of a chair with participants placed in a seated position with rigid back support, and hip and knee flexed at a 90° angle. Participants performed a brief warm‐up consisting of 2 × 5 sit‐to‐stand repetitions, from slow to fast pace, and lastly, 30 s of maximal effort sit‐to‐stand repetitions, following which participants were placed in the test chair. A seatbelt was fastened at the hips and a steel cuff was strapped around the distal lower leg approx. 3 cm above the medial malleoli, with the cuff connected to a strain‐gauge load cell via a rigid steel bar (Suetta et al., 2009). Lever arm length was measured from the knee joint axis of rotation (lateral femoral condyle) to the middle of the cuff (axis of rotation of the dynamometer).

For specific warm‐up, participants performed two sub‐maximal isometric knee extensor trials (at 50% and 80% max) before initiating the test. All participants performed 4–5 MVC trials interspersed by 45 s rest, following the instruction to contract as fast and forcefully as possible and maintain force exertion for approx. 3 s. Standardized verbal encouragement and visual online feedback provided by a real‐time display of the force output was given during all tests.

Data sampling was performed using an external 16‐bit analog‐to‐digital converter (NI‐USB‐6211, National Instruments Corp, Austin, USA), and all force signals were hardware amplified (National Instruments, Signal conditioning SC‐2345, Fullbridge SCC‐SG24) and subsequently sampled (1000 Hz), while during later off‐line analysis lowpass filtered with a zero‐lag 4th order Butterworth filter (20 Hz cutoff frequency).

In addition, rapid force capacity expressed as contractile RFD was determined in early phase (0–50 ms) as well as the late‐phase (0–200 ms) of rising muscle force (force onset *t* = 0) (Aagaard et al., 2002; Suetta et al., 2004). RFD was determined as the mean slope of the torque‐time curve in the pre‐defined time intervals (0–50, 0–200 ms, time 0 = onset of force) (Aagaard et al., 2002; Suetta et al., 2004). Trials with visible countermovement were discarded. Recorded force (i.e., torque) values normalized to body mass (kg) (Aagaard et al., 2002) along with absolute (i.e., non‐normalized) values were included in the subsequent analysis.

### 3.5 Statistical analysis

Descriptive data are presented as mean ± standard deviation (SD) unless otherwise stated and *p*‐values ≤0.05 were considered statistically significant. All analyses were made using the statistical software SigmaPlot (v. 13.0, Systat Software Inc, San Jose, CA).

All continuous data were visually inspected by plots of residuals, while also checked for normality using the Shapiro–Wilk test. Equal variance was ensured using the Brown–Forsythe test.

An unpaired two‐tailed *t*‐test was used to compare young and old participants for habitual activity levels. Between‐group (young vs. old) comparisons for non‐normally distributed data, were performed using a Mann–Whitney Rank Sum test. These data included blood analytes (eGFR and CRP) and activity count during the bed rest period (adherence to bed rest protocol).

Due to a lack of equal variance of insulin, HOMA‐IR, and stimulation intensities, these data were log‐transformed (log10) before the statistical analyses. Absolute values are presented.

To detect differences in response to bed rest (CON) and bed rest with electrical stimulation (NMES) a two‐way repeated‐measures ANOVA was performed, with the variables time (pre vs. post) and leg (NMES vs. CON). Further, for detection of differences between age groups, a two‐way repeated‐measures ANOVA on delta changes (%) from pre to post bed rest, with the variables leg (NMES vs. CON) and age group (young vs. old), was performed. A two‐way repeated‐measures ANOVA was used for analysis of anthropometric measures (body weight, BMI, fat‐free mass) and blood analyte concentrations, with the variables time (pre vs. post, or by day [0, 2, 4, 5]) and age group (young vs. old). When main effects or interactions were statistically significant (*p* ≤ 0.05), a Holm–Sidak post hoc analysis was performed.

## 4 RESULTS

### 4.1 Participant characteristics and changes in anthropometrics following 5‐day bed rest

Based on questionnaires related to daily activities and accelerometers, participants were categorized as recreationally active adults habitually engaged in low‐to‐moderate‐to‐high intensity physical activities before inclusion in the study (Table 1). There was no difference in habitual activity levels between young and old (activity level low: *p* = 0.0739, mid: *p* = 0.638, high: *p* = 0.850).

**TABLE 1:** Participant characteristics and habitual activity.

	Young (*n* = 16)		Old (*n* = 16)	
	Pre bed rest	Post bed rest	Pre bed rest	Post bed rest
Age (years)	25.3 ± 2.1		71.2 ± 3.3	
Height (cm)	179.1 ± 11.4		169.2 ± 9.2	
Weight (kg)	73.6 ± 14.0	72.7 ± 13.8	73.5 ± 10.3	73.1 ± 10.2
BMI (kg m^2^)	22.8 ± 2.2	22.5 ± 2.2	25.6 ± 2.3	25.5 ± 2.2
Fat‐free mass (kg)	51.35 ± 11.29	50.36 ± 11.21	47.92 ± 9.21	47.06 ± 9.07
Activity level (hours/day)				
Low	3.09 ± 1.15		2.43 ± 0.83	
Moderate	0.94 ± 0.72		0.80 ± 0.70	
High	0.13 ± 0.22		0.14 ± 0.16	

*Note*: Data are shown as mean ± SD. Activity levels were assessed based on questionnaires and activity sensors (accelerometers) placed on participants 3 days prior to the bed rest period. Main effect of time was detected for weight, BMI and FFM (*p* < 0.05) and old participants had a higher BMI compared to young (*p* < 0.05). No difference in activity levels between young and old was detected.

In general, participants' body weight, BMI, and fat‐free mass (FFM) were higher prior to bed rest than post bed rest (weight: *p* < 0.001, BMI *p* < 0.001, FFM *p* < 0.001), with old demonstrating higher BMI than young (*p* < 0.001) (Table 1).

On a group basis (young and old), there were no critical deviations from normal range levels in any of the analyte concentration levels in the comprehensive blood test panel, measured pre‐ and post‐bed rest period (supplementary data, Table S1).

### 4.2 Adherence to bed rest

During the 5‐day bed rest period, the accelerometer data indicated that while either in a sitting or supine position, young performed a total of 14.3 ± 8.8 min/day of low‐intensity lower limb movements, while old were active for a total of 21.1 ± 14.21 min/day (young vs. old, *p* = 0.213). These movements covered activities such as rolling onto the side, transitioning from a supine to a seated position, and transitioning from bed to wheelchair for toilet visits. No other activities for example standing or walking were registered during the bed rest period.

### 4.3 Blood analyte concentrations—Routine test panel

In the old, hemoglobin levels were within the normal range, but in the high end, before inclusion. At baseline, old had higher blood lipid concentrations than young (cholesterol HDL *p* = 0.006; cholesterol LDL *p* = 0.002, cholesterol *p* < 0.001), and cholesterol remained higher in old compared to young following bed rest (*p* = 0.013) (Table S1). Cholesterol levels increased following bed rest in young (*p* = 0.017) while decreased in old (*p* = 0.030). Further, cholesterol HDL decreased in both young (*p* = 0.004) and old (*p* < 0.001) following bed rest, while cholesterol LDL increased in the young only (*p* < 0.001) (Table S1).

As a general effect of time, increased levels of leukocytes (*p* = 0.001), and decreased levels of thrombocytes (*p* = 0.008) and Hb(B) hemoglobin A1c (*p* = 0.022) were detected, the latter being higher in old compared to young (*p* = 0.047).

C‐reactive protein (CRP) and Glomerular filtration rate (estimated, eGFR) were within the normal value range, except for one young participant, who had high CRP levels (pre: 25, post 32), but normal range values in all other variables.

Old had a higher eGFR level than young at pre (*p* < 0.001) and post (*p* < 0.001) bed rest. Two old participants had slightly elevated CPR levels (14 and 20 mg/L, respectively), which in the context of the other parameters were considered clinically irrelevant.

### 4.4 Glucose metabolism markers and insulin resistance index (HOMA IR‐index)

**FIGURE 2:**
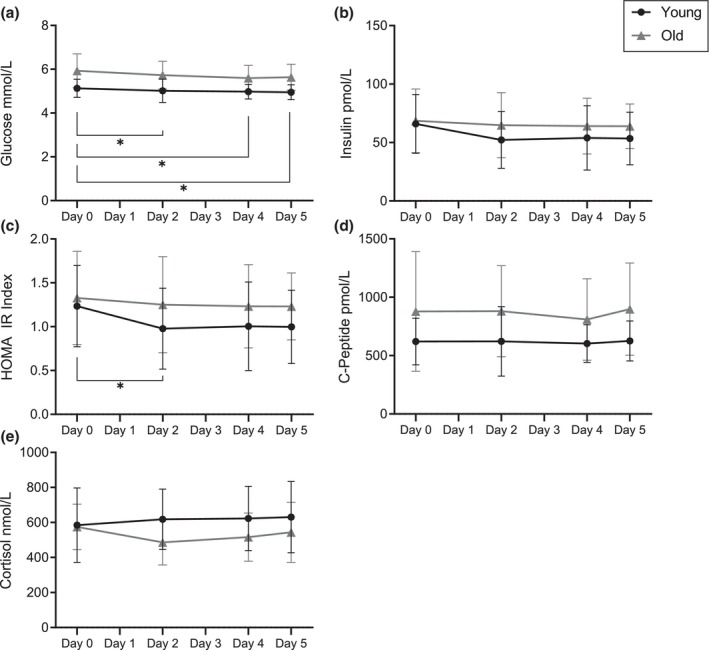
Changes in blood analyte concentrations and HOMA IR index during 5 days of bed rest. Data are presented as absolute values: Mean ± SD. *n* = 16 (young), 16 (old). (a) Changes in plasma glucose concentrations. (b) Changes in fasting insulin concentrations. (c) Changes in HOMA IR index. (d) Changes in fasting c‐peptide concentrations. (e) Changes in fasting cortisol concentrations. *Significant difference in concentrations between days (*p* < 0.05).

Generally, young demonstrated lower plasma concentrations of glucose and C‐peptide than old (*p* < 0.001, *p* = 0.018, respectively). Further, glucose levels decreased during the bed rest period, compared to Day 0 (*p* < 0.05) (Figure 2a). No differences nor changes were detected for cortisol or insulin (Table 2, Figure 2b,e).

**TABLE 2:** Changes in glucose metabolic markers and HOMA IR index following 5 days of bed rest.

	Day	Young (*n* = 16)	Old (*n* = 16)
Glucose (mmol/L)	Day 0	5.13 ± 0.41	5.93 ± 0.77
	Day 2*	5.02 ± 0.53	5.73 ± 0.63
	Day 4*	4.98 ± 0.33	5.60 ± 0.58
	Day 5*	4.95 ± 0.34	5.64 ± 0.59
Insulin (pmol/L)	Day 0	66.0 ± 25.1	68.5 ± 27.3
	Day 2	52.2 ± 24.3	64.8 ± 27.9
	Day 4	53.9 ± 27.5	64.1 ± 23.9
	Day 5	53.4 ± 22.5	63.9 ± 19.0
HOMA IR index	Day 0	1.24 ± 0.46	1.33 ± 0.53
	Day 2*	0.98 ± 0.46	1.25 ± 0.55
	Day 4	1.00 ± 0.51	1.23 ± 0.47
	Day 5	1.00 ± 0.42	1.23 ± 0.38
C‐peptide (pmol/L)	Day 0	620 ± 200	878 ± 513
	Day 2	622 ± 297	881 ± 390
	Day 4	603 ± 161	810 ± 350
	Day 5	626 ± 172	898 ± 395
Cortisol (mmol/L)	Day 0	585 ± 213	575 ± 130
	Day 2	618 ± 173	487 ± 129
	Day 4	623 ± 184	516 ± 138
	Day 5	631 ± 203	544 ± 172

*Note*: Values are mean ± SD.

* Significant difference from Day 0 (effect of time, young and old pooled, *p* < 0.05).

A main effect of time was detected for HOMA‐insulin resistance index (*p* = 0.029). Subsequent post hoc analysis revealed a decrease from Day 0 to 2 (*p* = 0.050) (Table 2, Figure 2c), accompanied by a tendency to decrease from Day 0 and 4 (*p* = 0.071).

### 4.5 NMES stimulation intensity

Young participants tolerated increased NMES stimulation intensity throughout the bed rest period from 25.2 ± 4.2 mA (Day 0) to 27.7 ± 4.5 mA (Day 1, + 10.3% from Day 0) to 30.8 ± 6.6 mA (Day 2, + 10.9% from Day 1) to 34.6 ± 7.6 mA (Day 3, +12.5% from Day 2) to 38.1 ± 9.1 mA (Day 4, + 10% from Day 3). Likewise, old participants demonstrated increased tolerance to NMES stimulation throughout the bed rest period, by increasing stimulation intensity from 31.9 ± 6.3 mA (Day 0) to 35.9 ± 8.4 mA (Day 1, +12.7% from Day 0) to 40.0 ± 10.5 mA (Day 2, + 11.3% from Day 1) to 43.7 ± 12.7 mA (Day 3, + 9.4% from Day 2) to 46.0 ± 14.1 mA (Day 4, + 5.1% from Day 3). Stimulation tolerance was significantly increased from day to day (increase Day 0 vs. 1 vs. 2: *p* < 0.001, increase Day 3 vs. 4: *p* = 0.013) within young and old, respectively. In general, old participants had an overall higher stimulation intensity than young (*p* = 0.005).

### 4.6 Muscle mass—Leg lean mass

**FIGURE 3:**
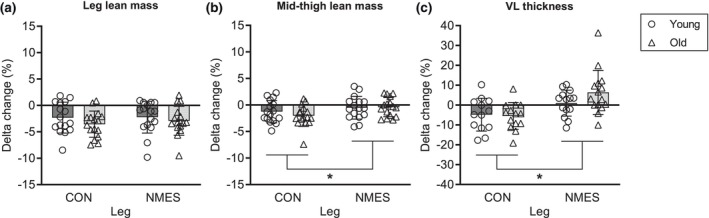
Percentage change from pre‐ to post 5 days of bed rest in muscle mass. CON, control leg, bed rest only. NMES: Stimulation leg, bed rest + daily NMES. Data are presented as mean ± SD with individual data points indicated. *n* = 16 (young), 16 (old) (a) changes in leg lean mass assessed by DXA scan. (b) Changes in mid‐thigh lean mass assessed by DXA scan. (c) Changes in vastus lateralis muscle thickness assessed by ultrasound imaging. *Significant difference between legs (*p* < 0.05). No difference between delta values (pre‐ to post bed rest) of young and old was detected.

Total leg lean mass decreased following bed rest in young (*p* = 0.004) and old (*p* < 0.001), respectively (Table 3), with leg lean mass in general being higher in NMES vs. CON in young (*p* = 0.025). Comparison of delta values (pre‐ to post bed rest) in LLM between young and old did not reveal any effect of NMES or age (Figure 3a).

**TABLE 3:** Changes in muscle mass following 5 days of bed rest.

	CON		NMES		Main effects		
	Pre	Post	Pre	Post	Time	Leg	Interaction
Leg lean mass (g)							
Young	8399 ± 1975	8226 ± 2033	8573 ± 2003	8400 ± 2038	***p* = 0.004**	***p* = 0.025**	*p* = 0.957
Old	7752 ± 1386	7482 ± 1388	7761 ± 1445	7531 ± 1393	***p* < 0.001**	*p* = 0.670	*p* = 0.296
Mid‐thigh lean mass (g)							
Young	664 ± 138	656 ± 137	680 ± 133	677 ± 133	*p* = 0.077	*p* = 0.081	*p* = 0.171
Old	619 ± 111	606 ± 107*	610 ± 107	607 ± 103	***p* = 0.002**	*p* = 0.586	***p* = 0.013**
VL thickness (cm)							
Young	2.36 ± 0.43	2.23 ± 0.37*	2.35 ± 0.34	2.37 ± 0.34	*p* = 0.262	*p* = 0.393	***p* = 0.021**
Old	2.14 ± 0.39	2.01 ± 0.33*	2.02 ± 0.36	2.14 ± 0.34*	*p* = 0.955	*p* = 0.681	***p* < 0.001**

Values are mean ± SD. Main effects are reported from a two‐way repeated measures ANOVA. *n* = 16 (young), 16 (old). CON: control leg, bed rest only. NMES: stimulation leg, bed rest + NMES. Leg lean mass and mid‐thigh lean mass (MTLM) were assessed by DXA and vastus lateralis (VL) thickness from ultrasound imaging. Bold values indicate significant of *p*‐values.

* Significant pre‐ to post bed rest changes within leg (*p* < 0.05).

### 4.7 Muscle mass—Mid‐thigh lean mass

In young participants, a tendency for an effect of time (*p* = 0.077) and leg (*p* = 0.081) was detected for MTLM (Table 3). In contrast, MTLM was preserved with NMES in old (*p* = 0.274) while found to decrease in CON (*p* < 0.001).

Comparing delta values (pre‐ to post‐bed rest) of MTLM between young and old, an effect of NMES was detected (*p* = 0.005), which was not age‐specific (Figure 3b).

### 4.8 Muscle thickness

Vastus lateralis muscle thickness was decreased following bed rest in young and old (*p* = 0.032; *p* = 0.014, respectively). In contrast, VL thickness remained unchanged following bed rest with NMES in young (*p* > 0.05) while increased in old (*p* = 0.012) (Table 3). No difference between NMES and CON was observed following bed rest for either young or old, while in old, a significant difference between intervention legs (CON vs. NMES) was detected pre‐bed rest (*p* = 0.047). The delta change in VL thickness was greater in NMES compared to CON (*p* < 0.001), with no difference between young and old (Figure 3c).

### 4.9 Maximal muscle strength

**FIGURE 4:**
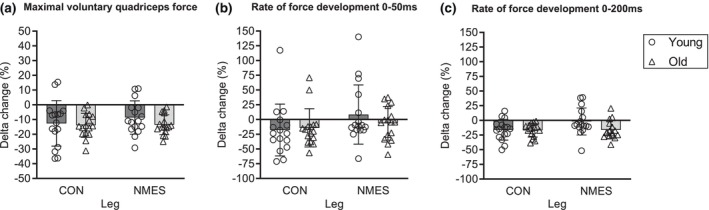
Percentage change from pre‐ to post 5 days of bed rest in mechanical muscle function. CON, control leg, bed rest only. NMES: Stimulation leg, bed rest + daily NMES. Data are presented as mean ± SD with individual data points indicated. *n* = 16 (young), 16 (old). (a) Percentage change in maximal muscle strength. (b) Percentage change in rate of force development (RFD) from 0 to 50 ms. (c) Percentage change in RFD from 0 to 200 ms. No difference between delta values (pre to post bed rest) of young and old was detected.

Knee extensor maximal muscle strength (MVC) strength normalized to body mass (Nm/kg) decreased in young (*p* = 0.002) and old (*p* < 0.001) from pre‐ to post‐bed rest, irrespective of NMES. As a main effect of leg, MVC strength (Nm/kg) was higher in CON legs than NMES legs in old (*p* = 0.041) (Table 4). Comparing delta values (percentage pre‐to‐post changes) in MVC between young and old, no effects of NMES or age could be observed (Figure 4a). Analyses of the non‐normalized data (Nm) yielded comparable results (Table 4).

**TABLE 4:** Changes in mechanical muscle function following 5 days of bed rest.

	CON		NMES		Main effects		
	Pre	Post	Pre	Post	Time	Leg	Interaction
Maximal isometric muscle strength (Nm/kg)							
Young	2.1 ± 0.6	1.8 ± 0.6	2.1 ± 0.7	1.8 ± 0.5	***p* = 0.002**	*p* = 0.962	*p* = 0.648
Old	1.5 ± 0.6	1.3 ± 0.5	1.4 ± 0.6	1.2 ± 0.5	***p* < 0.001**	***p* = 0.041**	*p* = 0.588
Maximal isometric muscle strength (Nm)							
Young	152.0 ± 53.1	132.2 ± 51.1	151.9 ± 64.7	133.4 ± 45.0	***p* = 0.003**	*p* = 0.918	*p* = 0.854
Old	130.2 ± 41.8	112.4 ± 35.6	127.3 ± 42.3	110.4 ± 36.7	***p* < 0.001**	*p* = 0.055	*p* = 0.511
RFD_0‐50ms_ (Nm/s/kg)							
Young	9.7 ± 4.1	7.44 ± 3.75	9.7 ± 5.5	9.3 ± 4.3	***p* = 0.022**	*p* = 0.198	*p* = 0.113
Old	7.1 ± 4.3	5.66 ± 3.58	6.8 ± 4.8	5.8 ± 4.4	***p* = 0.003**	*p* = 0.842	*p* = 0.369
RFD_0‐200ms_ (Nm/s/kg)							
Young	7.5 ± 2.6	6.2 ± 2.2*	7.3 ± 2.9	6.9 ± 2.52	***p* = 0.004**	*p* = 0.485	***p* = 0.022**
Old	5.6 ± 2.1	4.6 ± 2.1	5.2 ± 2.6	4.2 ± 2.0	***p* < 0.001**	***p* = 0.043**	*p* = 0.894

*Note*: Values are mean ± SD. Main effects are reported from a two‐way repeated measures ANOVA. *n* = 16 (young), 16 (old). CON: control leg, bed rest only. NMES: stimulation leg, bed rest + NMES. Bold values indicate significant of *p*‐values.

* Significant pre‐ to post bed rest changes within leg (*p* < 0.05).

### 4.10 Explosive force capacity (RFD)

RFD_50ms_ decreased in young (*p* = 0.022) and old (*p* = 0.003) from pre to post bed rest, irrespective of NMES (Table 4). No difference between young and old for delta values of RFD_50ms_ was detected (*p* > 0.05) (Figure 4b).

In young, RFD_200ms_ was preserved with NMES (pre vs. post: *p* = 0.259) while decreased in control legs (*p* < 0.001). Following bed rest, a tendency for a difference between legs (NMES vs. CON) was detected in young (*p* = 0.059) (Table 4). In contrast, RFD_200ms_ decreased in old following bed rest (*p* < 0.001). Generally, RFD_200ms_ was higher in CON vs. NMES (*p* = 0.043) (Table 4).

Comparing delta values of RFD_200ms_ between young and old, no effect of age was observed whereas in general, the delta change tended to be smaller in NMES compared to CON (*p* = 0.070) (Figure 4c).

## 5 DISCUSSION

As a main finding in the present study, daily sessions of NMES during short‐term 5‐day bed rest was effective of preventing muscle atrophy in the stimulated muscles of both young and old adults. Rapid force capacity (late‐phase RFD) was retained with NMES in young but not old participants, accompanied by decreases in early‐phase RFD (young and old) that were not prevented by NMES. Similarly, the protective effect of NMES did not seem to extend to maximal isometric muscle strength. Young and old participants responded similarly to both bed rest alone and bed rest with concurrent NMES, except for late‐phase RFD where a protective effect of NMES was observed only in young. Moreover, insulin sensitivity remained unaltered in both young and old during the bed rest period.

### 5.1 Adaptive changes in muscle mass following bed rest and NMES

Five days of bed rest led to significant reductions in whole‐leg muscle mass in the control limbs (i.e., non‐stimulated legs) of both young and old participants.

The observed decreases in leg lean mass of 2%–3.5%, are in line with previous reports of a 1%–4% reduction in leg lean mass in old participants following 5 days of bed rest (Reidy et al., 2017; Smeuninx et al., 2023; Tanner et al., 2015), and in young following 5 days immobilization (Wall et al., 2014).

Age‐specific atrophy responses have been reported following short‐term disuse, where a ~ 4% decline in lower limb lean mass was observed in older adults, but only negligible changes (−0.3%) were seen in younger adults (Reidy et al., 2018; Tanner et al., 2015). In contrast, others have demonstrated a 3% decline in thigh muscle CSA following 5–7 days of bed rest in young adults (Dirks et al., 2016; Mulder et al., 2015). Prior speculation has arisen regarding the muscle atrophy observed in young adults during immobilization (by cast/knee brace), as opposed to bed rest, and whether it can be attributed to the chosen disuse model (Tanner et al., 2015). Tanner et al. (2015) have suggested that the relatively more permissive conditions during bed rest, allowing for minor movements (e.g., knee bending, etc.), may be sufficient to slow down disuse‐induced atrophy in young adults. In the present study, we diligently ensured participants remained as motionless as possible with extended legs throughout the bed rest period. Consequently, we propose that the contrasting results in young adults, reported in the literature including the present results, are linked to the actual activity levels allowed during the respective disuse protocols.

Notably, MTLM was significantly reduced with bed rest in old CON legs, while only a tendency (*p* = 0.08) to change in MTLM was detected in young. This discrepancy may be attributed to variability and limited sensitivity in the assessment of regional muscle mass by DXA scan, as our ultrasound imaging revealed comparable reductions in muscle thickness for the CON leg of young and old participants after the bed rest period.

Given previous observations that muscular atrophy is initiated already in the very initial phase (days) of disuse (Hvid et al., 2014; Suetta et al., 2012; Wall et al., 2013) and that a large part of the observed decline in muscle mass can be attributed to the absence of muscular contractions (Maffiuletti et al., 2023), we expected that applying NMES to given muscle groups during a period of bed rest would counteract the magnitude of disuse atrophy, by replacing the lack of voluntary muscle activation by electrically stimulated contractile muscle activity.

As hypothesized, vastus lateralis muscle thickness was preserved in legs exposed to NMES, while reduced by ~5% in immobilized control legs (CON). These observations align with prior research reporting no change in thigh CSA following NMES during 5 days of one‐legged knee immobilization in young adults (Dirks et al., 2014) nor in thigh lean mass following 5 days of bed rest with concurrent NMES in old adults (Reidy et al., 2017). Notably, control groups (disuse only) exhibited a 4% reduction in both thigh CSA and thigh lean mass in the respective studies (Dirks et al., 2014; Reidy et al., 2017).

### 5.2 Adaptive changes in mechanical muscle function

In the present study, maximal voluntary muscle strength decreased by ~9–14% following 5 days of bed rest in both NMES and CON conditions irrespective of age. Albeit in contrast to our hypothesis, this is in line with previous reports of a ~ 7–15% decline in maximal knee extensor strength following 5 days of bed rest alone (Reidy et al., 2017; Tanner et al., 2015), and following 5 days of bed rest (Reidy et al., 2017) or one‐legged knee immobilization (Dirks et al., 2014) combined with concurrent daily NMES. The apparent mismatch between the preservation of muscle mass and MVC strength despite the involvement of NMES may, in part, be due to the non‐physiological mode of muscle activation with NMES, which deviates from patterns of voluntary muscle activation (Hortobágyi & Maffiuletti, 2011).

Research has demonstrated that higher stimulation intensities activate more profound parts of the muscle, in contrast to low‐intensity stimulation which predominantly activates the superficial part (Gravholt et al., 2023; Gregory & Bickel, 2005). Maximal stimulation intensity may, therefore, be a limiting factor for the activation of deeper muscle regions with NMES, although others have argued that superficial motor nerves can innervate myofibers deeper within the muscle, consequently reaching the profound parts of the stimulated muscles (Bickel et al., 2011). In the present study, NMES was performed using maximal tolerable stimulation intensities, with a progressive increase throughout the bed rest period. Despite this, and in line with others (Dirks et al., 2014; Reidy et al., 2017, 2020), we observed that maximal muscle strength decreased following bed rest irrespective of NMES intervention, and also despite that muscle thickness was preserved with NMES, which indicates that the deeper muscle compartments were activated by NMES.

As another novel finding in the present study, late‐phase RFD (0–200 ms) was conserved with NMES in young but not old following bed rest, while decreasing in non‐stimulated legs (CON), in agreement with previous reports of reduced levels of RFD following short‐term (3–4 days) disuse (Demangel et al., 2017; Hvid et al., 2014). Moreover, early‐phase RFD at 50 ms was observed to decrease following bed rest in both age groups, irrespectively of NMES.

Speculatively yet plausibly, the present observations may suggest that neural parameters, including maximal motor unit discharge rates and motor unit recruitment rates at force onset and in the subsequent phase of rising muscle force (0–200 ms), are negatively affected by short‐term disuse in young and old adults, resulting in preferential reductions in early‐phase RFD (0–50 ms), which is known to be strongly modulated by neural factors (Folland et al., 2014; Hannah et al., 2012; Maffiuletti et al., 2016). In this perspective, the present data may indicate a potential limitation of NMES intervention in counteracting the decremental effects of 5‐day disuse (bed rest) on the neural drive to myofibers during MVC efforts. Complicating this picture, signs of increases in neural drive in the form of enhanced evoked V‐wave responses during MVC have been reported following NMES intervention in young adults during free‐living (i.e., non‐disuse) ambulatory conditions (Gondin et al., 2006), suggesting that neuromuscular plasticity with NMES may differ between conditions of sustained disuse (strict bed rest) and free (i.e., non‐restricted) conditions.

We speculate that the present retainment in late‐phase RFD (RFD_200_) with NMES could be due to the stimulation/conservation of peripheral factors, such as muscle size, given that late‐phase RFD is strongly reliant on peripheral factors such as muscle mass, type 2 myofiber proportions and cross‐sectional area, and so forth. (Folland et al., 2014; Hannah et al., 2012; Maffiuletti et al., 2016). Furthermore, neural drive appears to be negatively affected (reduced) in old but not young adults in response to short‐term (days) disuse (Hvid et al., 2018), which may further have contributed to the retainment of RFD_200_ in young, but not old NMES legs. The observed reduction in late‐phase RFD in young CON legs possibly could be linked to the observed reduction in muscle thickness.

In our old participants, RFD_200_ was reduced in both CON and NMES legs, despite preservation of muscle thickness in NMES legs. As elaborated above we speculate this to be attributed to a potential decrease in neural drive in our old participants following bed rest, in turn offsetting the protective peripheral effect of NMES on muscle mass while leading to a net reduction in RFD.

### 5.3 Changes in glucose metabolism and insulin resistance

Insulin sensitivity (HOMA IR‐index) remained stable during the 5 days of bed rest. Decreased insulin sensitivity has been described as a phenotypic trait of muscular disuse (Crossland et al., 2019), as previous studies have reported increased whole‐body insulin resistance following 3–7 days of bed rest in young (Mikines et al., 1991; Reidy et al., 2018; Smorawiński et al., 2000; Stuart et al., 1988) and old (Reidy et al., 2018). In contrast, and in support of the present observations, Reidy et al. (2020) found no change in insulin sensitivity following 5 days of bed rest with or without NMES and protein supplementation in elderly participants (Reidy et al., 2020).

The constancy of the HOMA IR index with 5 days of bed rest could be attributed to the change from habitual diet to the controlled isocaloric diet provided during the days of bed rest. However, the present results may reflect an influence of NMES on the intricate relationship between insulin sensitivity and muscle inactivity, as also proposed in the literature (Dirks et al., 2016; Reidy et al., 2018).

### 5.4 Clinical implications

The findings of the present study emphasize NMES's potential as a therapeutic intervention tool during short‐term disuse for counteracting decline in muscle mass and partly muscle function. The observed preservation of muscle thickness with NMES during 5‐day disuse is crucial, considering the attenuated capacity for recovery observed in older adults after short‐term disuse (Hvid et al., 2014; Suetta et al., 2013). Moreover, the NMES equipment is both affordable and time‐efficient, facilitating its application to patients with minimal effort and allowing for management through periodic check‐ins rather than continuous supervision, thus enabling simultaneous treatment of multiple patients. However, further research is necessary to optimize specific stimulation protocols regarding targeted muscle groups, stimulus intensity, and session duration, thereby enhancing the potential for preserving functional abilities (e.g., chair rise, walking) and muscle mass with this therapy.

### 5.5 Conclusions

The present study demonstrates the effectiveness of daily NMES sessions during short‐term bed rest for preservation of knee extensor (VL) muscle thickness in young and old, while preventing a drop in late‐phase RFD in young but not old. In contrast, NMES failed to prevent a disuse‐induced decline in maximal knee extensor muscle strength in both young and old.

## AUTHOR CONTRIBUTIONS

*Conceptualization and design*: S.K.H., J.A., F.D., C.S. *Data acquisition and experimental work*: S.K.H., P.H., H.D.G., T.W.B., C.M.O. *Data analyses and interpretation*: S.K.H., P.H., H.N., H.D.G., T.W.B., P.A., L.G.H., J.A., F.D., C.S. *Writing*—*original draft*: S.K.H. *Writing*—*revision and approval of final version*: S.K.H., P.H., H.N., H.D.G., T.W.B., C.M.O., P.A., L.G.H., J.A., F.D., C.S. All authors agree to be accountable for all aspects of the work. All persons designated as authors qualify for authorship, and all those who qualify for authorship are listed.

## FUNDING INFORMATION

The project was supported by the Danish foundation helsefonden (22‐B‐0038).

## CONFLICT OF INTEREST STATEMENT

The authors have no conflict of interest to declare.

## Supporting information


**Table S1:** Changes in blood analyte concentrations pre‐post bed rest.

## Data Availability

All data generated and analyzed during this study are available from the corresponding author upon reasonable request. The data are not publicly available due to privacy or ethical restrictions.
