# Supplementary material for: Five days of bed rest in young and old adults: Retainment of skeletal muscle mass with neuromuscular electrical stimulation
Source: Physiol Rep. 2024 Aug 18;12(16):e16166. doi: 10.14814/phy2.16166 (PMC11330699; doi:10.14814/phy2.16166)
Supplement: Supplementary file 1 — Table S1: Changes in blood analyte concentrations pre‐post bed rest. [file PHY2-12-e16166-s001.docx]

|  | Young (n = 16) | |  | Old (n = 16) | | Main effects | | |
| --- | --- | --- | --- | --- | --- | --- | --- | --- |
|  | Pre bed rest | Post bed rest |  | Pre bed rest | Post bed rest | Time | Age | Interaction |
| **Hemoglobin**  (mmol/L) | 8.75 ± 0.74 | 9.03 ± 0.86 |  | 9.05 ± 0.81 | 8.99 ± 0.79 | p = 0.144 | p = 0.539 | p = 0.209 |
| **Leukocytes**  (x10^9^/L) | 5.62 ± 1.22 | 6.02 ± 1.92 |  | 5.38 ± 1.17 | 6.60 ± 1.67 | **p = 0.001** | p = 0.764 | p = 0.088 |
| **Thrombocytes** (x1000/µL) | 266.00 ± 74.42 | 254.38 ± 67.86 |  | 242.93 ± 48.70 | 233.53 ± 55.08 | **p =** **0.008** | p = 0.254 | p = 0.776 |
| **Hb(B) Hemoglobin A1c** (mmol/mol) | 34.00 ± 5.61 | 33.44 ± 5.85 |  | 37.44 ± 3.86 | 37.13 ± 3.88 | **p = 0.022** | **p = 0.047** | p = 0.495 |
| **Cholesterol**  (mmol/L) | 4.06 ± 0.61 | 4.40 ± 0.73* |  | 5.59 ± 1.13 | 5.25 ± 1.13* | p = 0.900 | **p < 0.001** | **p = 0.002** |
| **Cholesterol HDL** (mmol/L) | 1.45 ± 0.48 | 1.30 ± 0.47* |  | 1.92 ± 0.48 | 1.53 ± 0.37* | **p < 0.001** | **p =0.031** | **p = 0.002** |
| **Cholesterol LDL** (mmol/L) | 2.20 ± 0.53 | 2.66 ± 0.67* |  | 3.14 ± 0.97 | 3.11 ± 0.91 | **p = 0.008** | **p = 0.012** | **p = 0.010** |
| **Triglyceride**  (mmol/L) | 0.94 ± 0.56 | 0.96 ± 0.41 |  | 1.22 ± 0.60 | 1.33 ± 0.57 | p = 0.488 | p = 0.085 | p = 0.690 |
| **CRP**  (mg/L) | <1^§^ **/**  9.25 ± 15.20 | < 1^†^ **/**  9.67 ± 13.32 |  | < 1^#^ /  2.83 ± 1.47 | < 1^‡^ /  7.71 ± 7.23 |  |  |  |
| **eGRF**  **(**mL/min/1,73 m^2^) | > 90 | > 90 |  | 74.79 ± 11.99 | 69.86 ± 16.91 |  |  |  |

Values are mean ± SD. Main effects are reported from a two-way repeated measures ANOVA. CRP and eGFR are analyzed using Mann-Whitney Rank Sum Test. *Significant difference from pre- to post bed rest within group (young and old, p < 0.05). § n = 12 young participants. † n = 13 young participants. # n = 10 old participants.

‡ n = 8 old participants (of 15 old participants, one sample was missing).

**Table S1**. Changes in blood analyte concentrations pre-post bed rest
